# Single-dose rifampicin chemoprophylaxis protects those who need it least and is not a cost-effective intervention

**DOI:** 10.1371/journal.pntd.0006403

**Published:** 2018-06-07

**Authors:** Diana N. J. Lockwood, Padebettu Krishnamurthy, Bhushan Kumar, Gerson Penna

**Affiliations:** 1 Department of Clinical Research, London School of Hygiene and Tropical Medicine, London, United Kingdom; 2 Damien Foundation of India Trust, Chennai, India; 3 Department of Dermatology, STD and Leprosy, Postgraduate Institute of Medical Education and Research, Chandigarh, India; 4 Tropical Medicine Centre, University of Brasilia, Brasilia, Brazil; Hospital Infantil de Mexico Federico Gomez, UNITED STATES

We, as leprosy academics, have written the following letter to The Global Leprosy Programme and the Indian National Programme.

Single-dose rifampicin (SDR) treatment being offered to household contacts of new leprosy patients is being practiced by the Indian National Leprosy Programme since November 2017 [1]. We are concerned, because this is not an effective method for preventing multibacillary (MB) leprosy. It does not protect against the development of MB leprosy and does not protect immediate household contacts for a reasonable period of time. There are serious ethical problems related to identifying the contacts of patients with leprosy, it is not cost effective for household contacts, and there is a possibility of the widespread use of SDR promoting the development of rifampicin resistance genes in *Mycobacterium leprae*.

It is claimed that SDR gives 57% protection against the development of leprosy in contacts [2]. However, this is when all types of leprosy (paucibacillary [PB], MB, and single-lesion leprosy [SLL]) are combined. The main study testing the effectiveness of SDR in protecting against leprosy is the Chemoprophylaxis of Leprosy (COLEP) trial in Bangladesh [3]. In this study, 21,711 contacts of newly diagnosed leprosy patients were randomized to receive SDR or a placebo. Household contacts who took SDR did not have significant protection against developing leprosy (odds ratio [OR] 0.46 [0.15–1.38], *p* = 0.1652); it only protected neighbours of neighbours (OR 0.24 [0.11–0.52]) against the development of leprosy. SDR did not protect against the development of MB leprosy (0.52 [0.22–1.19], *p* = 0.1201); however, it did protect against the development of PB leprosy (0.38 [0.16–0.87], *p* = 0.0218) and single-lesion leprosy (0.42 [0.20–0.89]). Protection only lasted 2 years. These findings suggest that SDR treatment is only effective when patients have a low mycobacterial load, hence the protection only against the development of PB leprosy. In the nude mouse model, up to 20 doses of rifampicin 10 mg/kg were required to significantly decrease mRNA *M*. *leprae* levels in experimental leprosy, which again suggests that multiple doses of rifampicin will be needed if this intervention is to be effective [4]. One cannot assume that the index case is the source of infection to contacts in high endemic settings when there is a possibility of exposure to *M*. *leprae* from multiple resources.

SDR is being promoted because it is an easier intervention and any intervention that required 2 doses of rifampicin would be very challenging to administer. However, previous studies on leprosy chemotherapy have found that killing *M*. *leprae* often requires multiple doses of an active agent over several months.

A major benefit of a policy of giving SDR is that household contacts of leprosy patients will be examined. We know that these people are at highest risk of developing leprosy, so this is a good public health intervention. However, the ethical problems of identifying patients with leprosy when searching for the household contacts need to be explored carefully. The representatives of the patient association need to be consulted in a meaningful way. There is a risk that hasty implementation of this intervention could increase stigmatization by identifying patients with leprosy. The authors of the COLEP study found that 25% of people who refused to take chemoprophylaxis did so because of fears of having their leprosy status disclosed [5]. There are also ethical problems in telling people that they will be protected against the development of leprosy, because, if it happened, it would protect only some people from some types of leprosy and only for 2 years.

The intervention is least cost-effective for household contacts [6]. The Bangladesh study found that the cost of prevention of one case of leprosy was US$158, and preventive therapy was most effective in neighbours of neighbours, social contacts, and household contacts, in that order. The study also stated that to prevent the occurrence of one case of leprosy, 1,556 persons have to be treated. A large number of leprosy cases (135,485) were diagnosed in India in 2016 [7].

A recently published study from India found a delay in disease detection and institution of treatment long enough for children with leprosy to develop grade-2 deformity in significant numbers. It would be better to invest in improving case detection and diagnosis [8].

It is important to reiterate that poor coverage, which is common, may result in poor intervention efficacy. Isoniazid chemoprophylaxis in preventing TB, even though useful, is not implemented well; the coverage is less than 30% in India [9, 10]. If SDR had similar levels of coverage in real life with 57% efficacy and 30% coverage, the intervention efficacy would not be more than 20%.

Because SDR does not significantly reduce the number of patients with MB leprosy, it is unlikely to have an effect on transmission, because these are the patients that need to be treated earliest.

Another aspect that has not been satisfactorily addressed is the practical implications of giving SDR to people who also have concurrent TB infections. These may be either latent or fully manifest. This aspect has been discussed by an expert panel, but there were no data underlying the report [11]. The report did not make any clear recommendations as to how concurrent TB infections should be managed. Screening patients for TB infections is challenging in every setting.

Promoting rifampicin drug resistance in *M*. *leprae* may be a consequence of giving out a large amount of SDR. In 1982, WHO recommended that all leprosy patients should receive multidrug therapy [12]. This was done to prevent the emergence of rifampicin resistance. This has been a very successful policy. There has also been a successful shortening of the length of treatment. The low level of rifampicin resistance in *M*. *leprae* is very fortunate for the world of leprosy. This might be threatened by the widespread use of SDR as chemoprophylaxis. SDR will be given in thousands of doses to people with early leprosy. There is a potential risk that this will lead to the development of rifampicin-resistant *M*. *leprae*. If this occurs, the leprosy programme will be severely threatened. The proponents of SDR have considered the risk of rifampicin resistance developing in people with *M*. *tuberculosis* infections, but so far, the possibility of *M*. *leprae* resistance developing has not been considered [11]. This concern has been strengthened by the recent finding of genes coding for rifampicin resistance in *M*. *leprae* DNA isolated from biopsies taken from patients with both new and relapsed leprosy in several countries, including India and Brazil [13]. Resistance genes were found at a level of 3.8% in primary cases and 8% in cases who had relapsed after treatment.

Chemoprophylaxis is being implemented under the label of ‘Implementation research’ through the Leprosy Post-Exposure Prophylaxis (LPEP) Programmes. These are being implemented alongside national leprosy programmes in India, Indonesia, Myanmar, Nepal, Sri Lanka, Tanzania, Brazil, and Cambodia [14]. Contacts of ‘new cases’ diagnosed up to 2 years earlier are eligible [15]. After such a long interval, if the contact is not already showing signs of overt leprosy, surely, he/she is either not infected (therefore gaining no benefit) or is at a more advanced stage in subclinical disease, possibly with a higher bacterial load (less likely to respond to SDR). It would be better to design trials that test new interventions formally and will give us new information rather than implementing an intervention that gives some protection to some patients.

We urge the Indian programme and also the Global Leprosy Programme to withhold implementing this intervention and to instead develop research that identifies a more effective intervention. Research should also address the other issues that we have identified of confidentiality, cost-effectiveness, and the possibility of promoting the development of rifampicin-resistant *M*. *leprae*.
